# Journal of Clinical Monitoring and Computing end of year summary 2019: hemodynamic monitoring and management

**DOI:** 10.1007/s10877-020-00496-w

**Published:** 2020-03-14

**Authors:** Bernd Saugel, Lester A. H. Critchley, Thomas Kaufmann, Moritz Flick, Karim Kouz, Simon T. Vistisen, Thomas W. L. Scheeren

**Affiliations:** 1 Department of Anesthesiology, Center of Anesthesiology and Intensive Care Medicine, University Medical Center Hamburg-Eppendorf, Hamburg, Germany; 2 Outcomes Research Consortium, Cleveland, OH USA; 3 Department of Anesthesia and Intensive Care, The Chinese University of Hong Kong, Shatin, Hong Kong; 4 The Belford Hospital, Fort William, The Highlands Scotland, UK; 5 Department of Anesthesiology, University Medical Center Groningen, University of Groningen, Hanzeplein 1, 9700 RB Groningen, The Netherlands; 6 Department of Anaesthesia and Intensive Care, Aarhus University, Aarhus, Denmark

## Introduction

Hemodynamic monitoring is essential to provide optimal hemodynamic management to patients in perioperative and intensive care medicine. The Journal of Clinical Monitoring and Computing (JCMC) welcomes research articles investigating hemodynamic monitoring technologies, cardiovascular pathophysiology, and hemodynamic treatment strategies that help advance this research field and eventually improve patient care. In this review, we highlight and summarize selected papers on hemodynamic monitoring and management published in the JCMC in 2019.

## Blood pressure monitoring

In a prospective study, Nicklas et al. [1] compared a non-invasive continuous blood pressure monitoring system using the vascular unloading technique (CNAP system; CNSystems Medizintechnik AG, Graz, Austria) with standard intermittent oscillometric upper arm cuff blood pressure measurements with regard to the ability to detect hypotensive phases during complex gastrointestinal endoscopy. In 90 patients, the continuous blood pressure signal of the CNAP system was compared to intermittent oscillometric blood pressure set at five-minute intervals. The authors defined a hypotensive phase as a time period of ≥ 30 s with ≥ 50% of the CNAP blood pressure measurements at least 10% below the last oscillometric measurement with either concurrent mean arterial pressure (MAP) ≤ 65 mmHg or systolic arterial pressure (SAP) ≤ 90 mmHg. Twenty-six patients (29%) had hypotensive phases with low MAP and 30% with low SAP. Overall, continuous blood pressure monitoring using the vascular unloading technique was able to detect hypotensive phases earlier compared to intermittent oscillometric blood pressure measurements and also identified short hypotensive phases that would remain undetected between intermittent oscillometric measurements. The authors conclude that continuous non-invasive blood pressure measurement can help to detect intraoperative hypotension more rapidly and has therefore the potential to improve patient safety.

An observational cohort study in cardiac surgery patients was performed by Henriques et al. [2] to investigate the relationship between the complexity of preoperative blood pressure and pulse pressure (PP) and preoperative risk prediction using the Society of Thoracic Surgeons (STS) Risk of Mortality and Morbidity Index and the European System for Cardiac Operative Risk Evaluation Score II (EuroSCORE II). The complexity of blood pressure and PP was quantified using multiscaled entropy from time series extracted from the blood pressure waveforms. Additionally, time series measures, mean, and standard deviation (SD) were calculated. Data sets of 147 patients were included in the final analysis. The EuroSCORE II was calculated in all 147 patients, while the STS Mortality and Morbidity Index was only available in 115 patients (78%) having coronary artery bypass graft, aortic, or mitral valve surgery. Spearman correlation and linear regression were used to evaluate the relationship between blood pressure complexity and the risk indices. The results showed an inverse relationship between blood pressure complexity and the STS Morbidity and Mortality Index and EuroSCORE II. A one SD change in blood pressure complexity was associated with an increased risk for adverse outcome after cardiovascular surgery determined by either score. The results were consistent in a model adjusted for age, gender, and SD of the blood pressure time series. There are several limitations to this study, including that preoperative blood pressure measurements are only a rough estimate of normal baseline blood pressure [3], especially after premedication with midazolam. However, the results underline the importance of cardiovascular assessment before major surgery.

There is a risk of measurements artifacts in large data sets as measurements are saved electronically without controlling. A growing number of hospitals use electronic records in perioperative and intensive care medicine. Therefore, Du et al. [4] developed a new algorithm to identify measurement artifacts in automated records of perioperative blood pressure measurements. A total of 41,384 minute-by-minute blood pressure measurements of 54 anesthesia cases were analyzed and used to validate the developed algorithm against manual artifact identification. An error checking algorithm was applied to all blood pressure readings (SAP, diastolic, and MAP) to identify irregular recordings and subsequently replace them with linear interpolation of neighbors. Manual identification marked 509 blood pressure readings as artifactual, of which 443 were also identified by the new algorithm resulting in a sensitivity of 87.0%. The calculated specificity for the algorithm was 99.4%, as it marked 256 pressure readings as artifactual, which were marked as regular in manual analysis. In the original data set, 8.8% of the intraoperative MAP measurements were > 100 mmHg and 4.3% were < 55 mmHg. After automated and manual correction 7.3% (automated) or 7.3% (manual) of MAP measurements were > 100 mmHg and 2.0% (automated) or 2.1% (manual) were < 55 mmHg. The authors discuss potential limitations of the proposed algorithm during episodes with high blood pressure variability as it may be difficult to distinguish real variation from artifacts. Overall, artifact identification is an important issue and further development of error-checking algorithms may provide important support with data processing for researchers as well as clinicians using electronic medical records.

In a retrospective analysis, Harrison et al. [5] used the SaferSleep database with data from 55,896 pediatric anesthesia cases to report SAP alterations during cardiac and non-cardiac surgery. The authors included 2273 cases with intra-arterial blood pressure measurements and analyzed changes in intraoperative SAP in four different age groups. The patients were divided into the age-groups 1 to 30 days, 1 month to 1 year, 1 year to 5 years, and 5 to 6 years, and included all American Society of Anesthesiologists physical status classifications. The SAP changes were assessed over two measurement intervals, 30 s and 300 s, and analyzed using normalization and principal component analysis. The mean (SD) SAP in age-group 1 to 30 days old was 55.7 (16.7) mmHg during cardiac surgery and 55.2 (17.6) mmHg during non-cardiac surgery. Age-group 1 month to 1 year had a mean SAP of 64.8 (23.2) mmHg during unspecified cardiac surgery and 70.6 (21.6) mmHg during non-cardiac surgery. The mean SAP in patients 1 year to 5 years old was 68.6 (23.5) mmHg during cardiac surgery and 80.1 (18.8) mmHg during non-cardiac surgery. In the age-group with 5 to 6 years, the mean SAP was 71.8 (24.8) mmHg during cardiac surgery and 85.4 (17.7) mmHg during non-cardiac surgery. The average changes in SAP over 30 s and 300 s were similar across all groups and both cardiac and non-cardiac cases. The presented data do not distinguish between different methods for general anesthesia, and only SAP was included. Additionally, the age-group 1 to 5 years old included a heterogeneous group of pediatric patients. The authors concluded that further research on hemodynamic management of pediatric patients is necessary to provide a better understanding of their cardiovascular physiology and the response to general anesthesia. This is an important research topic and the current study provides interesting information on the variation of blood pressure in different age-groups.

## Blood flow monitoring

In 2019, five papers on cardiac output monitoring were published in the JCMC.

Vetrugno et al. [6] compared transpulmonary thermodilution cardiac output (VolumeView/EV1000/Hemosphere; Edwards Lifesciences, Irvine, CA, USA) to pulmonary artery thermodilution cardiac output (pulmonary artery catheter and Vigilance/Hemosphere monitor; Edwards Lifesciences) in 49 patients undergoing liver transplant surgery. The percentage error ranged from 29% to 43% depending on the stage of surgery, data being least in agreement during the anhepatic and reperfusion stages. Trending ability was poor even within the defined stages of surgery. One would have expected better agreements and the authors’ conclusions did not reflect the wide variations in readings between the two methods, especially during the anhepatic and reperfusion stages. Based on these findings, the use of the VolumeView/EV 1000/Hemosphere system during liver transplantation surgery could not be recommended.

Also using the Volume View/EV 1000/Hemosphere system, Nakwan et al. [7] investigated the validity of two cardiac contractility indices derived from the transpulmonary thermodilution curve, (i) cardiac function index (CFI) and (ii) global ejection fraction (GEF). Transthoracic echocardiography was used as reference method, measuring left ventricular ejection fraction. Thirty-two ventilated septic shock patients were included and receiver operating characteristics (ROC) curves were used to calculate the predictive ability of the CFI and GEF for the left ventricular ejection fraction. The results showed an area under the ROC curve (AUC_ROC_) for the CFI to predict a left ventricular ejection fraction ≥ 40% (AUC_ROC_: 0.926), ≥ 50% (AUC_ROC_: 0.924), and ≥ 60% (AUC_ROC_: 0.875). Similar results were found for the predictive ability of the GEF (left ventricular ejection fraction ≥ 40% (AUC_ROC_: 0.934), ≥ 50% (AUC_ROC_: 0.938), and ≥ 60% (AUC_ROC_: 0.887). Further studies were recommended to confirm these findings.

Maeda et al. [8] compared cardiac output values obtained with the fourth generation FloTrac/Vigileo software (Edwards Lifesciences) to pulmonary artery catheter thermodilution-derived readings. Cardiac output by the Fick Method using the E-CAiOV (GE Healthcare, Chicago, IL, USA) was also included in the study. Data were collected from 22 cardiac surgery patients at six time points. Systemic vascular resistance was increased by administering phenylephrine. The data comparisons were notable because of the appalling percentage errors of over 70% for the Fick Method with the E-CAiOV, though percentage error did improve to < 30% for the FloTrac when cardiac output was high.

One of the more recently marketed technologies to measure cardiac output is the BSM-9101 bedside monitor (Nihon Kohden, Tokyo, Japan). It provides estimated continuous cardiac output (esCCO) based on pulse wave transit time technology measured using the electrocardiogram (ECG) and peripheral pulse oximeter. Suzuki et al. [9] compared both cardiac output and stroke volume variation (SVV) against FloTrac readings in 21 post cardiovascular surgery patients, mainly aortic aneurysm repairs. The data did not support the clinical use of esCCO as percentage errors were 69% and 99% for cardiac output and SVV, respectively. However, arterial pulse contour analysis is not an established reference method in cardiac output validation studies. Furthermore, choosing a patient cohort with significant arterial disease further challenges the reliability of the pulse contour method. Despite the authors’ optimism about their results, one has to question the soundness of the study design.

Tantot et al. [10] studied the monitoring potential of an index derived from the ratio of real-time PP over MAP (PP/MAP). The index was used intra-operatively to guide fluid or vasopressor administration. One hundred and three neurosurgery patients were studied. The rationale was the similarity of PP/MAP to the Liljestrand–Zander equation which is used to transform the arterial pressure wave into blood flow measurements. This equation has been shown to track vasopressor induced changes in cardiac output in several studies. Oesophageal Doppler (i.e. CardioQ, Deltex Medical, Chichester, UK) was used as the reference method. The best correlation between variation in PP/MAP and changes in cardiac output was found after phenylephrine (r = 0.53, p < 0.001) and norepinephrine (r = 0.41, p < 0.001). However, the results after ephedrine infusion or fluid administration showed no correlation. The authors’ overall conclusion was that monitoring PP/MAP would be difficult to apply in current clinical practice.

## Cardiovascular physiology

Arterial stiffness is a prognostic variable for cardiovascular morbidity and mortality [11]. Changes in arterial stiffness can be assessed by measuring the changes in pulse wave velocity (PWV). Two systems which are generally used in clinical practice to measure PWV are Biopac (Biopac Systems Inc, Goleta, CA, USA) which uses a photoplethysmography sensor placed on the fingertip combined with ECG leads, and Complior (Alam Medical, Vincennes, France) which uses piezoelectric sensors placed on the skin over the carotid and radial arteries. In the April issue, Van Velzen et al. [12] compared these two systems in healthy volunteers, which were subjected to various positional changes, and found that the Biopac system measured consistently and significantly lower PWV values compared to the Complior system. In seated healthy volunteers, the Biopac system measured a PWV of 3.0 ± 0.2 m/s compared to a PWV of 10.2 ± 1.4 m/s for the Complior system. The Biopac system measures a more peripheral trajectory with the vessels of the finger being included. More peripheral vessels are narrower and possibly more compliant, which likely reduces PWV. The authors discuss that these factors may outweigh the reduced radius of the peripheral vessels, which potentially increases PWV. The correlation coefficient between the PWV values of the two systems and Bland–Altman analysis of the measured PWV values showed that there was a fair agreement between the two methods. The authors conclude that both systems can be used to measure changes in PWV as long as the difference in magnitude between the systems is taken into account. Clinicians wanting to measure a singular PWV for diagnosis and prognosis should be aware that the absolute PWV values may differ considerably based on the method used.

Capillary refill time (CRT) is the time for skin color to fully return after applying firm pressure at the index finger. CRT is part of the clinical examination of the critically ill patient suspected of circulatory shock as it is a variable of peripheral tissue perfusion. However, there may be inter-observer variability for the measurement of CRT as it is a subjective assessment. Optical infrared spectroscopy can be used to objectively measure peripheral tissue perfusion (this is referred to as blood refill time (BRT) to distinguish the two methods). Optically measured BRT rather than visually assessed CRT allows for a high fidelity mechanical measurement of peripheral blood perfusion. At the moment, there is uncertainty regarding the potential interference of temperature and age on measurements of peripheral tissue perfusion [13]. In the April issue, Shinozaki et al. [14] performed measurements of BRT in healthy volunteers during different temperature settings and found that a lower fingertip temperature significantly increased BRT. A mechanical device was built to objectively measure BRT, which compresses and releases the fingertip in a standardized manner, and estimated changes in blood volume using infrared light. At room temperature, the volunteers’ fingertip temperature was 32.1 ± 3.0 °C, with a BRT of 1.96 (95% confidence interval (CI) 1.60–2.33) seconds. When the volunteers’ hands were submerged in 15 °C water, the fingertip temperature was significantly colder (23.6 ± 3.6 °C), with a significant increase in BRT to 4.67 (95% CI 3.57–5.76) seconds. Different age and race of the healthy volunteers were analyzed as possible confounders, but after adjusting for the temperature difference, the sample size proved to be too small to provide reliable conclusions. One of the findings of this article is that clinicians should be careful in the interpretation of bedside measurements of peripheral blood perfusion, as these are influenced by temperature.

Phenylephrine is an α1-adrenergic receptor agonist that is predominantly used to increase vascular tone, and thus increase cardiac afterload and blood pressure [15]. There is still uncertainty regarding the effects of phenylephrine on venous return, preload, and contractility. Wodack et al. [16] assessed the hemodynamic effects of phenylephrine in an animal study and found that phenylephrine increases effective preload and contractility besides its known vascular properties. The authors investigated these effects in eight mechanically ventilated pigs, which were subjected to a step-wise increase in phenylephrine dosages. Hemodynamic data were obtained after placing an arterial catheter, central venous catheter, aortic and pulmonary artery flow probes, and a transpulmonary thermodilution monitor (PiCCO2; Pulsion Medical Systems, Feldkirchen, Germany). The measured MAP was used to define baseline MAP (M0; 62 ± 7 mmHg), and a MAP increase of 50% (M1; 87 ± 5 mmHg) and 100% (M2; 112 ± 4 mmHg) were chosen to define the relevant time points. The preload enhancing effects of phenylephrine were demonstrated by an increase in global end-diastolic volume (GEDV): GEDV was 362 ± 51 mL at M0 and increased to 405 ± 72 mL at M1 and to 415 ± 58 mL at M2. The increased contractility with phenylephrine administration was demonstrated by an increase in CFI, GEF, and the aortic dPmx, which is a variable to estimate pressure changes during the systolic phase. CFI was 7.1 ± 1.1 L/min at M0 and increased to 8.5 ± 1.1 L/min at M1, and to 9.7 ± 1.6 L/min at M2. GEF was 33.3 ± 5.7% at baseline and increased to 38.5 ± 3.5% at M1, but did not increase further at M2 (39.2 ± 4%). dPmx was 628 ± 285 mmHg/s at baseline and increased to 1105 ± 535 mmHg/s at M1, and to 1607 ± 758 mmHg/s at M2. The authors conclude that phenylephrine may be investigated in clinical practice as measure to recruit preload and increase contractility.

The article discussed above was accompanied by an editorial discussing the physiology involved in phenylephrine-induced recruitable preload. In this editorial, Jacobs et al. [17] discuss the promising results shown in the animal study by Wodack et al. [16] and elaborate on the potential role of the early application of vasopressors to recruit blood from the venous compartment. The authors first revisit the contemporary standards of fluid resuscitation in patients with circulatory shock, which find their origin in the aggressive fluid treatment applied in patients with cholera almost 200 years ago. We now know that care has to be taken to prevent excessive fluid administration, as this leads to edema and increased morbidity in critically ill patients [18]. Second, the cardiovascular physiology involved with the application of vasoconstriction to recruit preload is discussed. Furthermore, the results obtained in the animal study are revisited, and potential limitations of the study design are discussed. The authors of the editorial conclude that the early application of vasopressors has promise, but more clinical studies are needed to evaluate how these management changes may affect patient outcomes.

The assessment of right ventricular dysfunction has gained increased interest in perioperative and intensive care as it is associated with mortality and organ dysfunction. Vistisen et al. [19] performed a retrospective analysis with data from the Medical Information Mart for Intensive Care III database from Beth Israel Deaconess Medical Center [20] to investigate characteristics of the waveform of post-extrasystolic beats in patients with right ventricular dysfunction and healthy controls. The authors identified 24 patients with right ventricular dysfunction and 34 control patients with available echocardiographic reports. The right ventricular function was analyzed in the 2nd and 3rd post-ectopic beat after ventricular extrasystoles. The mean reduction of the SAP at the 2nd and 3rd beat was lower in the group with right ventricular dysfunction compared to the control group with a mean (SD) of −1.7 (1.9)% vs. −3.6 (1.9)%. The AUC_ROC_ for the identification of right ventricular dysfunction based on reduction in SAP was 0.76 with an optimal specificity of 91% and a corresponding sensitivity of 50%. Using maximization of the Youden index, the respective specificity was 71% with a sensitivity of 75% at a threshold of -2.85%. Even though extrasystoles and the analysis of concurrent hemodynamic changes may be of great interest for analysis of the cardiovascular system, they occur infrequently and are only observed in few patients. Therefore, monitoring devices would have to detect extrasystoles and subsequent changes in SAP. The accompanying editorial by Pinsky [21] discusses the study by Vistisen et al. [19] in detail underlining the importance of right ventricular function and its assessment in critical care patients.

An experimental method comparison study with 12 Yorkshire pigs was performed by Monge Garcia et al. [22] to evaluate the estimation of arterial elastance from different sites in comparison with arterial elastance measured with a left ventricular conductance catheter as reference method. The authors obtained arterial blood pressure measurements from aortic, femoral, and radial artery catheters. The calculation of arterial elastance was based on arterial pressure with arterial elastance based on 90% of SAP, MAP, and dicrotic notch pressure in relation to the stroke volume. During the study period, several maneuvers were performed to induce changes in afterload (phenylephrine and nitroprusside), preload (bleeding and fluid bolus), and contractility (esmolol and dobutamine). Bland–Altman analysis was performed to compare the agreement between the different estimates of arterial elastance. The overall results showed good correlation between arterial elastance measurements obtained with the different arterial catheters (aortic, femoral, radial) and different methods (90% SAP, MAP, dicrotic notch) with the reference method (all r^2^ > 0.92). The calculation of arterial elastance based on MAP (MAP/stroke volume) showed the lowest mean of the differences and narrow limits of agreement. The authors support the use of MAP as a standard for the calculation of arterial elastance, since the measurements were interchangeable between the different measurement sites. With increased interest in arterial elastance as a measurement for arterial load this study provides interesting evidence for future research in this field.

An observational study in 15 cardiac surgery patients was performed by Tusman et al. [23] to evaluate, whether the photoplethysmography (PPG) signal can be used to detect changes in arterial blood pressure and vascular tone. The systemic vascular resistance and vascular compliance (stroke volume/PP) were used as surrogates for vascular tone. Based on the amplitude of the PPG signal (maximum to minimum) and the position of the dicrotic notch, seven classes of vascular tone were defined. A dicrotic notch between 20 and 50% of the amplitude was defined as normal vascular tone (Class III). A decreased PPG amplitude and a dicrotic notch near the systolic peak (Class I) or in the upper 50% of the PPG waveform (Class II). An increased PPG amplitude and a dicrotic notch at 20% of the maximum of the PPG waveform (Class IV), at the foot of the PPG (Class V) or even a negative dicrotic notch (Class VI) were used to identify states with vasodilation. A total of 190 datasets with 61 hypertensive (vasoconstriction), 84 normotensive, and 45 hypotensive (vasodilation) episodes were included in the final analysis. A Spearman rank test showed a correlation between the PPG based classification of vascular tone with SAP (r =  −0.90, p < 0.0001), systemic vascular resistance (r =  −0.72, p < 0.0001), and vascular compliance (r =  −0.77, p < 0.0001). The analysis of only the amplitude of the PPG signal showed a correlation with SAP (r =   −0.79, p < 0.0001), systemic vascular resistance (r = −0.66, p < 0.0001), and vascular compliance (r = 0.82, p < 0.0001) as well. Overall, a total of 183 out of 190 episodes were correctly identified as hypertensive, normotensive, or hypotensive. The calculated sensitivity was 100% with a specificity of 97.9% for the detection of hypotension and 94.9% with a specificity of 99.2% for the detection of hypertension. The authors concluded that changes in arterial blood pressure mediated by changes in vascular tone were closely connected to the shape of the PPG waveform. However, future studies will need to investigate the accuracy and applicability in a broader patient spectrum.

In another prospective observational study, Li et al. [24] used a porcine cardiac arrest model to investigate if the PPG waveform can identify the return of spontaneous circulation (ROSC) during cardiopulmonary resuscitation. Six pigs received chest compressions without cardiac arrest and six pigs had three minutes of untreated ventricular fibrillation followed by two minutes with chest compressions and subsequent defibrillation. After ROSC, chest compressions were performed for another five minutes. ROSC was defined as a measurable pulse and blood pressure with an abrupt increase in end-tidal carbon dioxide (≥ 40 mmHg). Time and frequency domain methods were used to analyze the PPG waveform. At baseline both groups showed a single peak in the frequency domain method. In the group without cardiac arrest the results showed a stable heart rate, whereas diastolic blood pressure increased and end-tidal carbon dioxide decreased during chest compressions compared to baseline. At the same time, the time domain method showed a hybrid fluctuation or “envelope” phenomenon with a “double” or “fusion” peak in the frequency domain method. Interestingly, one of the peaks had a rate around 110 per minute matching the rate of chest compressions, while the second peak matched the “drifting” frequency of a spontaneous pulse in the group without cardiac arrest. In the group with ventricular fibrillation, the PPG waveform disappeared during ventricular fibrillation, but returned after the initiation of chest compressions. After defibrillation, the PPG waveform showed a hybrid fluctuation or envelope phenomenon with double or fusion peak similar to the group without cardiac arrest. The authors state that these effects may be caused by chest compressions with simultaneous regular pulse and depend on the frequency deviation. Therefore, the authors argue that the existence of two peaks may be a characteristic of ROSC during cardiopulmonary resuscitation. However, future research is necessary to investigate the clinical applicability of this method.

## Perioperative goal-directed therapy

Perioperative goal-directed therapy (pGDT) is a widely adopted strategy for optimizing cardiovascular dynamics in perioperative medicine. pGDT has been shown to improve postoperative outcomes in patients having high-risk surgery of different kinds and has been implemented in several national and international guidelines. Nevertheless, its implementation into clinical practice is not widespread, and even in places where it is used, protocol adherence is hampered by workload, cost issues related to additional monitoring, and skepticism of clinical staff. The consequence is a large inter- and even intra-provider variability in hemodynamic management [25], resulting in varying outcomes [26]. This issue may be resolved by offering clinicians a real-time decision support helping them to decide when and how much volume and vasoactive agents should be given to optimize the patient’s hemodynamics. Joosten et al. [27] tested the feasibility of the commercially available “Assisted Fluid Management” (AFM) software (Edwards Lifesciences) to guide pGDT in 46 patients undergoing major abdominal surgery. The software was build based on algorithms previously created and validated by the same group of authors for developing a closed-loop fluid administration system. It is primarily based on SVV but also considers heart rate, MAP, and stroke volume, and is “learning” from the individual patient’s hemodynamic reaction on the successive fluid administrations. When the AFM software suggests giving a fluid bolus, this suggestion could be followed or not by the provider. If the fluid option was chosen, a button on the interface screen was pushed so that the system could analyze the hemodynamic status before and after fluid administration. In the current study, the time spent below predefined targets of SVV as well as the fluid requirements of these patients was compared to those of a historical control group of 38 patients receiving conventional pGDT with the same hemodynamic targets. Maintenance infusion was low (2 mL/kg/h) in both groups, and 250 mL of crystalloids were supposed to be given when SVV >13% in the control group or when the AFM software suggested so in the intervention group. In addition, vasopressors were allowed to treat hypotension (defined as MAP < 65 mmHg) not related to hypovolemia, and colloids in case of major blood loss (> 1000 mL). The authors tested the hypothesis that AFM increased the time spent with a SVV < 13% as compared to pGDT (control). And indeed, patients in the AFM group spent more time below the target SVV than the historical control group (median 92% vs. 76%, p < 0.0005), despite receiving less fluid (1775 mL vs. 2350 mL, p = 0.01). The resulting less positive fluid balance (1010 mL vs. 1725 mL, p < 0.001), however, did not translate into improved patient outcomes, as postoperative complications were similar between groups. Yet, the length of stay in the intensive care unit (ICU) or post anesthesia care unit and in hospital were significantly shorter in the AFM group. The AFM software recommended 5 fluid administrations on average per patient (range 0–18), i.e. about one per hour during surgery. A high proportion of the recommended fluid administrations (238/245; 97%) were followed by the clinicians, of which 52–65% lead to an increase in stroke volume of 15% and 10% or more, respectively. Unfortunately, protocol compliance was not registered in the control group. The high compliance in the AFM group might be explained by the plausibility of the recommendations to give fluids, meeting the clinicians’ expectations, and the reduced workload.

In the accompanying editorial comment, van Beest [28] discussed the difficulties of implementing new strategies into clinical practice. Furthermore, he pointed out that although both AFM and the conventional pGDT algorithm were based on SVV in the Joosten study [27], actually the AFM algorithm might have been more physiologically driven by learning from the individual reactions to previous fluid administrations. He was convinced that AFM might alleviate the implementation of pGDT protocols into clinical practice.

A different approach of guiding pGDT was chosen by Cesur et al. [29] in their prospective randomized study. The pleth variability index (PVI) is a non-invasively derived (from pulse oximetry) dynamic variable to predict fluid responsiveness based on heart–lung interactions [30] that has previously been used to guide pGDT [31]. Cesur et al. compared a PVI-guided pGDT (using a PVI threshold of > 13% to trigger a 250 mL colloid administration) with a conventional fluid management (CFM; based on blood pressure, heart rate, central venous pressure (CVP) and urine output) in 70 patients having colorectal surgery. Of note, the maintenance fluid rates differed markedly (2 mL/kg/h in the pGDT group vs. 4–8 mL/kg/h in the CFM group). The primary endpoints were fluid requirements as well as lactate and creatinine levels. Fluid administration was significantly higher in the CFM group (1950 vs. 900 mL, p < 0.001), while fluid balance was significantly lower in the pGDT group (620 vs. 1400 mL, p < 0.001). Again, these differences did not translate into outcome differences besides a slightly shorter time to passage of stool in the pGDT group (4.5 vs. 5 days). The other endpoints, such as renal function and length of hospital stay, were also similar in both groups. This study confirms previous findings showing that dynamic variables were superior to static variables like CVP to guide fluid management, and that these dynamic variables can be derived non-invasively by pulse oximetry.

Besides hemodynamic monitoring, which is an integral component of pGDT, also depth-of-anesthesia (DoA) monitoring might contribute to the outcome of high-risk surgical patients. In a before-after study setup, Lima et al. [32] studied the effect of implementing guidelines for hemodynamic and DoA monitoring in their hospital on intraoperative drug and fluid consumption and postoperative complications. They retrospectively included a large number of patients (n = 596) undergoing abdominal cancer surgery, 313 of which were included before (before group) and 283 after protocol implementation (after group) after having instructed their caregivers on the hemodynamic and DoA targets of their guidelines. While baseline characteristics and procedures were comparable between groups, postoperative morbidity (particularly delirium and urinary tract infections) was significantly lower and hospital length of stay shorter after guideline implementation. This was associated with an about 50% increase in the use of hemodynamic and DoA monitoring and a reduction in intraoperative fluid administration. Interestingly, the adoption of the new monitoring guidelines varied across specific variables, with DoA monitoring being implemented in most patients (88%), cardiac output monitoring in 61% and central venous oxygen saturation monitoring in only 29%. The authors speculated that invasiveness, costs, and unease of use may have hampered the use of hemodynamic monitoring in their study. The fact that they did find outcome benefits despite the low protocol implementation rate implies that the effects might have been even larger with a higher adoption of hemodynamic optimization.

The advantages and disadvantages of a before-after study design as opposed to the scientific standard of a randomized controlled trial were discussed in the accompanying editorial comment by Saugel et al. [33]. While the question about the best study design to investigate a potentially beneficial impact of pGDT on patient outcome cannot be definitely answered, the editorial comes to the conclusion that both study designs have their place and value in contributing to find out if pGDT actually improves patient outcome in real-life daily clinical practice.

## Fluid responsiveness

Measuring dynamic fluid responsiveness variables normally requires advanced hemodynamic monitoring. The smartphone application Capstesia (Galenic App, Vitoria-Gasteiz, Spain) is able to calculate pulse pressure variation (PPV) from a picture of the invasive arterial pressure waveform taken from any monitor screen without the need for additional hemodynamic monitoring systems or sensors. Joosten et al. [34] compared PPV obtained with Capstesia with SVV measured with uncalibrated pulse wave analysis in 40 patients with major abdominal surgery based on predefined categories (PPV and SVV < 9%, 9–13%, and > 13%) reflecting decision thresholds to administer fluids. The overall agreement between PPV and SVV was 79% and the Kappa coefficient was 0.55. After the induction of general anesthesia but before surgical incision, an accuracy of 84% and a Kappa coefficient of 0.61 were observed. In only 1% of the cases, PPV and SVV would have resulted in completely opposite clinical decisions. The authors concluded that Capstesia might be an easy and usable alternative to advanced monitoring technologies for the assessment of dynamic fluid responsiveness variables. Further studies are needed to confirm the potential and limits of this application.

Park et al. [35] investigated another method to predict fluid responsiveness in 38 pediatric patients having cardiac or neurosurgical surgery using respiratory variations in the pulse oximetry plethysmography waveform (ΔPOP). Since it has been shown that the contacting force between the sensor and measurement site affects the signal quality, this study investigated the effect of different contacting force conditions on ΔPOP and the ability of the system to predict fluid responsiveness. The ability of ΔPOP to predict fluid responsiveness before a 10 mL/kg 6% Volulyte (Fresenius Kabi GmbH, Bad Homburg, Germany) infusion was significantly different between fluid responders and non-responders at a contacting force of 0.9–1.2 N (AUC_ROC_: 0.815 [95% CI 0.674–0.956; p = 0.002]) and at individually adjusted contacting force (AUC_ROC_: 0.847 [95% CI 0.716–0.978; p < 0.000]). Since ΔPOP seems to be a reliable indicator to predict fluid responsiveness at a certain contacting force and is measured non-invasively and relatively easy, this study is an important contribution for this methodology.

Another interesting and clinically relevant study in 88 patients with spinal surgery investigated the ability of PPV to predict fluid responsiveness in prone compared to supine patient position and studied the effects of body mass index, intra-abdominal pressure, and respiratory system compliance (CS) on PPV [36]. The authors measured PPV, intra-abdominal pressure, and CS in the supine position, after changing into the prone position, and after a fluid challenge of 500 mL isotonic saline in the prone position. The study revealed that PPV in the prone position was able to predict fluid responsiveness compared to PPV in the supine position if the patient’s body mass index was < 30 kg/m^2^ and CS > 31 mL/cmH_2_O. Since the prone position is obligatory for many surgical procedures and a treatment option in patients suffering from acute respiratory distress syndrome, this study provides clinically important insights for these indications.

Vistisen et al. [37] investigated the use of extrasystoles and micro-fluid challenges to predict fluid responsiveness as an alternative to PPV in two different time windows (after anesthesia induction and during bypass preparation) in 61 patients having elective coronary artery bypass graft surgery. In each time window, after an initial observation for extrasystoles, a micro-fluid challenge (50 mL in 10 s) was performed and thereafter a traditional fluid challenge (5 mL/kg). The study revealed insufficient validity of the two investigated methods during cardiac surgery, which might have been caused by several factors (e.g., study setting, patients’ demographic characteristics, premedication with beta-blockers) or simply a non-adequate predictive ability of the methods in these specific patients. Since dynamic variables such as PPV and SVV have several limitations (e.g., special ventilator settings, non-usability during open-chest surgery) this study is an important contribution to find alternatives for predicting fluid responsiveness in patients with unreliable PPV measurements. However, new methods for predicting fluid responsiveness have to be investigated further, especially to define their area of application and limitations.

In a pilot study, Pybus [38] investigated the feasibility of performing real-time spectral analyses of the respiratory and arterial pressure waveform in 60 cardiac surgical patients and assessed the clinical utility of this technique to predict fluid responsiveness. For this purpose, he performed real-time calculation of the “spectral peak ratio” (SPeR) in patients undergoing aortic valve replacement during an increase of the tidal volume over 2 min and found a strong linear correlation between SPeR and tidal volume. The slope β of this relationship may be used to represent the slope of the cardiac response curve at its equilibrium point with the venous return curve in the “classical” Guyton model, and changed significantly after aortic valve replacement (1.58 ± 0.78 vs. 1.79 ± 0.8). Additionally, β fell significantly during a passive leg raising maneuver. Further studies are needed to evaluate this technique in detail and find its place in daily clinical practice.

Sun et al. [39] performed a prospective method comparison study to evaluate the agreement of the pulse amplitude variation assessed with PPG at the finger and on the forehead with PPV derived from an arterial blood pressure signal. A total of 29 patients having major vascular or urologic surgery were included in the study. Bland–Altman analysis showed a mean of the differences between finger derived pulse amplitude variation and PPV of 3.2 ± 5.1%, which improved to 1.2 ± 3.8% after baseline correction. The forehead-derived pulse amplitude variation had a larger difference of the means with 12.0 ± 9.1%, which decreased to 3.3 ± 4.8% after baseline correction. The results indicated no effect of other potential confounding factors like the heart rate to respiratory rate ratio, the perfusion index, and the PPV itself. Overall, the results indicated that pulse amplitude variation may serve as a non-invasive alternative to PPV. However, the PPG signal may be insufficient in critical care patients with shock or on high-dose vasopressor therapy as peripheral perfusion may be impaired. Additionally, pulse amplitude variation needs detailed analysis of the pulse wave and may not bet detected by eyeballing.

## Artificial intelligence and machine learning

The number of published studies applying machine learning methods to clinical data is exploding these years, and for JCMC, 2019 was also mirroring this trend. Last year, we saw five original papers applying various types of machine learning techniques/algorithms to clinical data. All studies had a retrospective design and authors predominantly tried to predict hemodynamic events/derangements such as tachycardia [40], hypotension [41] and cardiac arrest [42], but we also saw one before/after implementation study [43] and another study trying to identify patterns between clinical practice and outcomes advised by machine learning techniques [44]. For one of the original papers [40], an accompanying editorial was published highlighting important aspects of the reporting of such papers [45]. Finally, general and future aspects of applying machine learning to continuously monitored physiological data was highlighted in an excellent narrative review [46], where Rush et al. stated in their introduction that “Machine learning is a term likely spoken of more than understood. Machine learning is most simply defined as the use of various statistical techniques that can be employed to make predictions and decisions based on similarities in what is being analyzed to what has previously been observed.” The review gave a nice state-of-the-art overview and pointed out aspects of what still needs to be done to set free the potential of applying machine learning to physiological data. A key point was that we rarely see true machine learning, in the sense that most of the implemented models are static models that do not continue to learn from collected data. The authors defined this a hybrid format of machine learning, because machine learning is used to develop the (finally) static model. The authors discussed the role of machine learning and also acknowledge other artificial intelligence algorithms such as physiologic modelling. But, no matter the algorithm, human clinical knowledge will always remain necessary in the care of patients [46]. The authors reviewed specific clinical monitoring problems, where machine learning may play a role such as identification of sepsis, delirium, and ventilator dyssynchrony or reduction of false alarms and sedation management. At the time of writing the review, none of the reviewed methods had provided clear indications for a clinical benefit, but this probably reflects the negligible pool of prospective validation studies likely to emerge in coming years. The authors finally discussed various perspectives of the future implementation of machine learning algorithms and what obstacles need to be addressed. These include the imperative aspects of false alarms, technological development for hospital IT-infrastructure to support real-time implementation of algorithms, and the need for model explainability for clinicians (as opposed to black-boxes) to understand and perhaps even “adhere to” e.g., predictions made by a machine learning model. As discussed by the authors, this may require a new type of clinician with a strong trans-disciplinary knowledge and mindset. The review is highly recommended reading!

In line with the aspects of the review, the editorial by Vistisen et al. [45] accompanying the paper by Yoon et al. [40] also highlighted the need for sensible methodology and clear reporting of studies applying machine learning to physiologic data. Yoon et al. [40] developed a model to predict tachycardia based on 1-min trending values of vital signs such as heart rate, blood pressure, respiratory rate, and spectral features of these. Based on 787 episodes of tachycardia (cases) and 705 control periods without tachycardia (non-cases), the authors predicted tachycardia with an AUC_ROC_ of 0.81 with their developed algorithm. The editorial discussed how to identify the data sets for cases and non-cases, a selection that is fundamentally acausal if the existence of an event is used to define the data set, i.e. if analyzing only selected temporal windows preceding events and non-events, which Yoon et al. [40] did. In turn, one would expect a dramatically different performance of such an algorithm, once set free in a prospective validation study, likely associated with an unacceptably high false positive rate, i.e. false alarms [45]. Also, the handling of correlated features is important to consider along with the choice of prediction model. Finally, model evaluation is important, not least to compare the model performance with that of a simple reference model, such as carry-forward classification, which in this case would be heart rate, since the prediction concerned tachycardia (but would be e.g., blood pressure if the prediction was concerning hypotension). Yoon et al. [40] chose a sensible modelling approach, where correlated features should be handled well. Still, some expectedly highly correlated features seemed to remain in the final model, which theoretically may be suboptimal, and the authors refrained from comparing with a simple carry-forward model.

The study by Donald et al. [41] presented a Bayesian artificial neural network approach for predicting hypotension in neurocritical care patients suffering traumatic brain injury. The authors felt that the algorithm worked well, even though the AUC_ROC_ was only in the range of 0.7, which is not as good as that reported for existing predictive monitoring [47]. An important aspect, however, is that Donald et al. [41] were working on a more difficult cohort, likely to present with severe and/or paroxysmal autonomic problems. The way the authors predicted hypotension was also different, e.g., predictions were generally made early with respect to hypotensive events (15–20 min) and based on other hypotension definitions and therefore difficult to compare with previous research [47]. Regarding the aspects suggested to be reported for such a study [45], it appeared that no classification comparison was made with a simple carry-forward model, i.e. blood pressure itself. Also, the authors created their dataset in the same acausal way described above. Yet, the authors were very much aware of the possible problems of false positive classification and should be commended for taking the first steps in order to improve that possible issue.

Matam et al. [42] described a machine learning based framework to predict cardiac arrest in the pediatric ICU. The authors presented an extremely well annotated data set, where several auditing steps were taken to confirm the correctness of the labels defining ground truth—cases and non-cases of cardiac arrest. Also, the authors correctly stratified the modelling on age groups because pathophysiology of hemodynamic features (such as heart rate) change from newborns to teenagers, which the data set spanned. The features used for the prediction was derived from normal vital signs, i.e. heart rate, respiratory rate, blood pressure, saturation (both 125 Hz waveform data and 5 s trending data). The machine learning approach was somewhat different from the regular understanding of the term machine learning, where a training set—features from both cases and non-cases – is fed to a learning algorithm that seeks to differentiate between the two case groups. In the study, an auto-regressive model was developed, in which non-cases were modelled. Afterwards, that model was presented with a balanced and matched set of 69 cases and 69 non-cases and the algorithm predicted cardiac arrest with a AUC_ROC_ of around 0.75 10 min in advance of events. The peripheral oxygen saturation signal seemed to provide similar classification (AUC_ROC_ of 0.77), so the more advanced combination of vital signs in this group of patients for this predictive purpose may not be very beneficial. This highlights the need for reporting comparative classification of simple/existing monitoring [45]. After the model development and classification, the authors had clinicians to audit the data set to define which cardiac arrests were clinically possible to predict and compared their classification with that. They highlighted that only few (6%) cardiac arrest events were judged clinically predictable, whereas their algorithm predicted 91% of these events. This may be on the expense of specificity, and since the data selection appeared acausal in this study, false alarms may be an issue to handle in a future, prospective validation study, which we hope the authors will conduct.

JCMC also published a validation study that reported clinical outcome data before and after the implementation of the Continuous Monitoring of Event Trajectories (CoMET) system (Advanced Medical Predictive Devices, Diagnostics, and Displays, Charlottesville, VA, USA) in a surgical ICU [43]. The CoMET system visualizes an underlying algorithm’s estimation of cardiorespiratory instability. Ruminski et al. [43] estimated the occurrence of different clinical outcomes 7 months before and 7 months after clinical implementation: septic shock development (sepsis-II definition), hemorrhage, respiratory failure, and ICU mortality. The authors showed that the rate ratio (before/after ratio of events indexed to patient days in the ICU) of septic shock was significantly lower in the “after implementation” period. A total of 34 patients developed septic shock in the before period and only 17 did so in the after period. In a control cohort from a medical ICU, similar changes were not seen. These data seem promising, but should still be treated with some caution. First, the study is not a randomized controlled trial, and before/after studies, particularly those that are retrospective, hold potential for various types of biases. For instance, a randomized controlled trial would always be prospectively defined and virtually always be announced in a trial registry in advance of the trial, declaring primary and secondary outcomes, analysis plans, etc. It would have been a convincing asset, if this before/after study had been prospectively defined. Choices of outcomes and their definitions (e.g., choosing to index number of events to length of ICU stay) and other aspects, for instance the choice of 7 (and not e.g., 12) months defining before and after periods would then be more clear. Before/after studies also need to evaluate how patients were included and excluded. An important aspect of the inclusion for the “septic shock analysis” is that approximately 134 patients out of 840 (16%) were identified with shock on ICU admission in the before period. In the after period, this figure was around 200 patients out of 907 (22%). These (statistically highly significant) disproportions may be something to consider as a possible selection bias, because these patients were excluded from subsequent analysis and could therefore for instance not “develop” a septic shock in the analyzed cohorts. Looking at crude absolute number of events, a “reduction” of 17 septic shock cases (from 34 to 17) was observed. However, ICU mortality still “increased” with 15 cases (from 55 to 70), which was not explained by slightly different cohort sizes, so the point estimate of all-cause mortality increased – which sure enough could also be biased by the different rates of shock-on-admission. While definitely applauding the authors for taking the next step of validation of machine learning algorithms, firm conclusions are inherently difficult to draw from retrospective before/after studies as compared with more controlled designs.

In the study by Maheshwari et al. [44], the authors segmented a multicentric clinical dataset of 1786 patients having colorectal surgery. The segmentation was done with topographical data analysis, which can group patients in an advanced way based on the various features from the dataset, while maintaining a transparent and interpretable reporting/visualization of which features drove the segmentation. The segmentation algorithm identified nine distinct groups and saw that clinical treatments such as ketorolac use and less intra-operative fluid were associated with the best clinical outcomes. The association between fluid administration and clinical outcome is not new, which the authors recognized. The authors also stated that their tool is neither meant to establish causality in any way, nor as state-of-the-art epidemiological tool. The system is a very strong tool for providing feedback about clinical practice and its variation to individual clinicians, clinical teams, and the clinical administration. If data and the analysis from such a system is interpreted with a clear understanding of what biases may impact associations, it would likely be a valuable tool to provide a foundation for an internal, clinical discussion about e.g., adherence to treatment guidelines such as enhanced recovery after surgery programs. This is possible given the transparent nature of the system and a nicely highlighted perspective of the authors.

In summary, machine learning techniques gain widespread interest and utilization. JCMC not only publishes but also receives an increasing number of papers applying machine learning to hemodynamic and clinical data. The field is still new but exciting for authors, editors, and reviewers and we look very much forward to seeing which impact all these efforts provide in future, preferably prospective, studies. Studies need sufficient reporting [45] as well as discussion of various bias types [46], particularly if studies are retrospective, which the vast majority of published papers still are. Potential biases in the underlying (retrospective) data are difficult to identify and evaluate when advanced and hard-to-interpret machine learning algorithms are applied to them.

## Technical developments

In an in-silico study, Rinehart et al. [48] evaluated the performance of a closed-loop vasopressor controller during different levels of norepinephrine responsiveness. The authors have previously [49] shown acceptable functionality with varying degrees of infusion line delay and altered pharmacokinetics. A physiologic Monte-Carlo simulation was used to simulate 3500 random septic patients with seven different levels of norepinephrine response ranging from 0.1 × to 10 × with 1 × as reference for a usually expected response. Additionally, the simulated patients were separated into having stable and dynamic sepsis through the application of a stable or randomly changing “shock factor” to mimic vasodilation. The performance of the controller for each level was evaluated through the median performance error and median absolute performance error, wobble analysis, and time out of specific target ranges. The median performance error was less than 5% and the wobble was below 3% for all response levels. The divergence was negative, but decreased towards zero at the highest (10x) response level resulting in oscillating blood pressure levels. The authors explain this phenomenon with the controller not being able to make small enough adjustments. In contrast, the time out of target range was significantly longer at the lowest norepinephrine response levels of 0.1 × and 0.2 × compared to usual norepinephrine response due to decreased correction speed as the controller slowly adjusted infusion rate. Overall the results are promising and the authors conclude that the closed-loop vasopressor controller remained effective despite different norepinephrine response levels. This is an important finding and shows that the investigated closed-loop controller is able to adjust for unknown physiological variations. Research in this field has made great progress in the last years and automated or semi-automated infusion of vasopressors, fluids as well as anesthetics may support our daily work in the operating room and ICU in the future.

A nationwide survey was conducted by Scholten et al. [50] to investigate the role of ultrasound in the current practice for the placement of central venous catheters among anesthesiologists and intensivists in the Netherlands. All Members of the Dutch Society of Anaesthesiology (Nederlandse Vereniging voor Anesthesiologie) and the Dutch Society for Intensive Care (Nederlandse Vereniging voor Intensive Care) were invited to participate in the survey. The survey included questions regarding physician and hospital characteristics, the use of ultrasound as well as the NEO Five-Factor Inventory-3 (NEO-FFI-3), a questionnaire to evaluate personality domains (extraversion, openness to experience, agreeableness, and conscientiousness). A total of 506 out of 2,291 (22%) participants completed the survey and were included in the analysis. Out of these 506 participants, 68% reported that ultrasound was used always or almost always for the placement of central venous catheters. The authors identified “working in a non-academic non-teaching hospital”, “providing cardiac anesthesia”, and “more years of physician experience” as factors associated with less frequent use of ultrasound guidance after multivariate analysis. The most cited reasons for not using ultrasound were the fear for loss of landmark skills (28.6%), a lack of ultrasound equipment (22.7%), and no perceived benefit over landmark method (20.9%). Two thirds of the study participants reported that a complication occurred during central venous catheter placement in the past year at their department. 43% of these complications occurred despite use of ultrasound guidance. Only 13% of the participants had never experienced a complication during central venous catheter placement. The evaluation of the NEO-FFI-3 showed only a minor association of neuroticism and extraversion with the use of ultrasound guidance suggesting that personality traits only play a minor role in the use of ultrasound guidance. The authors conclude that providing evidence supporting the use of ultrasound might not be sufficient to increase regular use of ultrasound guidance for central venous catheter placement and that change in local protocols is necessary and can improve patient safety.
